# A Comparative Study on the Effect of Substrate Structure on Electrochemical Performance and Stability of Electrodeposited Platinum and Iridium Oxide Coatings for Neural Electrodes

**DOI:** 10.3390/mi15010070

**Published:** 2023-12-29

**Authors:** Linze Li, Changqing Jiang, Luming Li

**Affiliations:** 1 School of Mechanical Engineering and Automation, Fuzhou University, Fuzhou 350108, China; 2 National Engineering Research Center of Neuromodulation, School of Aerospace Engineering, Tsinghua University, Beijing 100084, China; 3 IDG/McGovern Institute for Brain Research, Tsinghua University, Beijing 100084, China

**Keywords:** hierarchical structures, femtosecond laser, platinum, iridium oxide, neural electrodes, coating stability

## Abstract

Implantable electrodes are crucial for stimulation safety and recording quality of neuronal activity. To enhance their electrochemical performance, electrodeposited nanostructured platinum (nanoPt) and iridium oxide (IrO_x_) have been proposed due to their advantages of in situ deposition and ease of processing. However, their unstable adhesion has been a challenge in practical applications. This study investigated the electrochemical performance and stability of nanoPt and IrO_x_ coatings on hierarchical platinum-iridium (Pt-Ir) substrates prepared by femtosecond laser, compared with the coatings on smooth Pt-Ir substrates. Ultrasonic testing, agarose gel testing, and cyclic voltammetry (CV) testing were used to evaluate the coatings’ stability. Results showed that the hierarchical Pt-Ir substrate significantly enhanced the charge-storage capacity of electrodes with both coatings to more than 330 mC/cm^2^, which was over 75 times that of the smooth Pt-Ir electrode. The hierarchical substrate could also reduce the cracking of nanoPt coatings after ultrasonic, agarose gel and CV testing. Although some shedding was observed in the IrO_x_ coating on the hierarchical substrate after one hour of sonication, it showed good stability in the agarose gel and CV tests. Stable nanoPt and IrO_x_ coatings may not only improve the electrochemical performance but also benefit the function of neurobiochemical detection.

## 1. Introduction

Neural electrodes can be used to stimulate neural tissues such as the brain, vagus nerve, and spinal cord, having effectively treated Parkinson’s disease [1], drug-resistant epilepsy [2], and restored patients’ walking after paralysis [3]. With developments in the treatment, the electrodes’ sizes are decreasing and their applications are extending (such as visual prostheses [4,5], and nerve blocking by direct current [6,7]). To meet the requirements of these developing techniques, electrodes must allow the injection of higher charge while maintaining low impedance [8,9]. Although platinum and platinum-iridium alloys have been used clinically for a long time, their limited range of charge injection has hindered the extending applications [4,6].

Various advanced materials have been proposed to enhance the electrochemical performance of neural electrodes [8,9]. Hydrogels and conductive polymers possess lower elastic moduli and superior electrochemical properties. But they face challenges such as coating delamination and inadequate long-term stability [10]. Carbon materials like carbon nanotubes and graphene exhibit electrochemical stability, capacitive electrochemical behavior, a wide electrochemical window, and fast electron transfer kinetics [9]. However, there are still issues with their batch preparation processes and long-term biocompatibility demonstration [9]. Noble metals or metal oxides are crucial materials for clinical applications due to their remarkable chemical inertness and corrosion resistance [11]. Electrodeposited metal and metal oxide coatings, including platinum [12] and iridium oxide [13,14,15], have attracted attention due to their ease of processing and the ability to be added in situ. Additionally, recent studies have shown that electrochemically deposited platinum [12] and iridium oxide [13] coatings exhibit good biocompatibility. However, their adhesion stability is a critical issue for practical applications.

The methods used to enhance the stability of platinum and iridium oxide coatings can be broadly categorized into two approaches. The first approach involves using chemical methods to alter the bonding strength between the substrate and the coating. For instance, incorporating one or more polydopamine adhesion layers has been shown to improve the stability of platinum black coatings on gold under ultrasonic testing [16]. However, the introduction of adhesion layer materials may also introduce potential biocompatibility concerns. The second approach employs physical methods to enhance coating adhesion by creating the mechanical anchoring of the surface micro-nanostructure. Electrochemical roughening of the platinum electrode with sulfuric acid has been shown to enhance the stability of the platinum and iridium oxide coatings on platinum under repeated cyclic voltammetry cycles [17]. Nevertheless, corrosive reagents are needed in this method. Femtosecond laser structuring has emerged as an important surface modification technique for different materials due to its ultrashort pulse and ultrahigh energy intensity. Recent studies have demonstrated that micro-nanostructures created by femtosecond laser can enhance the coatings’ adhesion and improved their resistance to friction [18], tensile [19], and shear [20]. For neural electrodes, femtosecond laser direct writing technology has also been utilized to prepare hierarchical structures on the clinically commonly used platinum-iridium alloy (Pt-Ir), which can highly enhance the electrochemical performance of the electrodes [21,22]. The use of Pt-Ir with hierarchical structures as a substrate presents a promising avenue to improve not only the coating stability but also electrode performance. The effect of substrate structure on the electrochemical performance and stability of platinum and iridium oxide coatings deserves investigation.

In this study, nanostructured platinum (nanoPt) and iridium oxide (IrO_x_) coatings were electrochemically deposited on femtosecond laser-prepared hierarchical platinum-iridium and smooth platinum-iridium substrates. The influence of the substrate structure on the electrochemical properties and stability of the electrode coatings was explored through ultrasonic, agarose gel and cyclic voltammetry testing.

## 2. Materials and Methods

### 2.1. Electrode Fabrication

**Figure 1:**
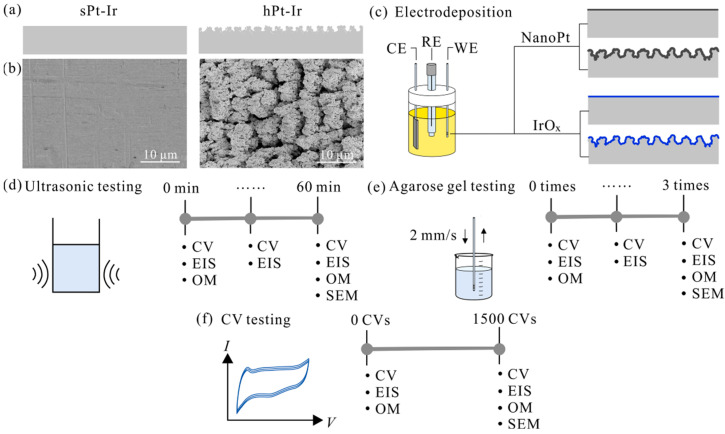
Study overview. (**a**) Cross-sectional schematic diagram and (**b**) surface morphology under scanning electron microscope (SEM) of smooth platinum-iridium (sPt-Ir) and hierarchical platinum-iridium (hPt-Ir) substrates. (**c**) Electrodeposition of nanoPt and IrO_x_ coatings on sPt-Ir and hPt-Ir substrates. WE, RE, and CE represent working electrode, reference electrode, and counter electrode, respectively. (**d**) Ultrasonic testing, (**e**) agarose gel testing, and (**f**) cyclic voltammetry (CV) testing. EIS: electrochemical impedance spectroscopy. OM: optical microscopy.

The Pt-Ir electrodes were fabricated according to established protocols [21,22]. Bare PtIr10 alloy tubes (composed of 90% Pt and 10% Ir) with a diameter of 1.3 mm were used. Hierarchical Pt-Ir (hPt-Ir) substrates were created using a femtosecond laser system by helix line scanning with optimized parameters including repetition rate, pulse energy, and scanning speed [21]. In order to further improve the electrochemical performance, the scanning interval was adjusted from 8 μm to 6 μm [22]. The smooth Pt-Ir (sPt-Ir) substrates were directly cut from the untreated Pt-Ir tube. The lengths of both sPt-Ir and hPt-Ir substrates were 1.5 mm, and their cross-sectional schematic diagram and surface morphology are shown in Figure 1a,b. Prior to electrodeposition, all electrodes were rinsed in isopropanol and deionized water and dried.

### 2.2. NanoPt and IrO_x_ Coating Preparation

The nanoPt and IrO_x_ coatings were prepared by electrodeposition method on both sPt-Ir and hPt-Ir electrodes using an electrochemical workstation (CHI 660E, CHI, Shanghai, China) with a three-electrode setup (Figure 1c). The working electrode was either an sPt-Ir or hPt-Ir electrode, the reference electrode was an Ag/AgCl electrode filled with a saturated KCl solution (Model 218, Leici, Shanghai, China), and the counter electrode was a titanium sheet (20 mm × 30 mm× 0.3 mm). NanoPt coating was prepared from an aqueous solution of 5 mM H_2_PtCl_6_ at a rate of 120 mV/s scanning between −0.3 V and 0.3 V for 180, 360, 540, and 720 cycles [12]. The IrO_x_ coatings were electrodeposited in Ir electrolyte (4 mM IrCl_4_, 1 wt% H_2_O_2_, 40 mM (COOH)_2_·2H_2_O, and adding potassium carbonate to bring pH to ~10.5) at a potential of 0.55 V versus Ag/AgCl for a duration of 15, 30, 45, and 60 min [14]. 

### 2.3. Electrochemical Performance Characterization

The electrochemical performance of the electrodes was evaluated using the same electrochemical workstation and three electrode configuration as the electrodeposition process. Cyclic voltammetry (CV) and electrochemical impedance spectroscopy (EIS) were performed in 0.01 M 1× PBS solution (Solarbio, Beijing, China). The CV measurements were carried out by scanning the potential from the open circuit potential with a range of −0.6 V to 0.8 V versus the reference electrode at a scan rate of 50 mV/s. The charge-storage capacity (CSC) was obtained by integrating the cathodic current density over time. For EIS testing, a 10 mV root-mean-square sinusoidal signal with zero direct current offset was applied, and the frequency was swept from 0.1 Hz to 100 kHz.

### 2.4. Surface Morphology Characterization

The surface morphology of the coatings was examined using a scanning electron microscope (SEM, Helios G4 CX, FEI, Hillsboro, OR, USA) with a working voltage of 5 kV. To gain insights into the cross-sectional structure of the coatings and substrates, focused ion beam (FIB) slicing was carried out using the same equipment. Optical microscope (OM) with a CCD camera was also used for inspection before and after testing.

### 2.5. Surface Element Analysis

X-ray photoelectron spectroscopy (XPS) was used to analyze the surface element of smooth and hierarchical platinum-iridium substrates before and after adding nanoPt (deposited by 720 CV cycles) and IrO_x_ coatings (deposited by 60 min).

### 2.6. Ultrasonic Testing

The mechanical stability of the nanoPt (deposited by 720 CV cycles) and IrO_x_ coatings (deposited by 60 min) on sPt-Ir and hPt-Ir substrates was tested in water using an ultrasonic bath (150 W, KQ3200E, Kunshan Shumei, Kunshan, China) as illustrated in Figure 1d [15,16,23]. The number of samples was three for the different coatings on different substrates. OM inspection was conducted before testing. The CV and EIS measurements were carried out at the timepoint of 0, 15, 30, and 60 min during testing. After the testing, the coating morphology was examined using SEM and OM. 

### 2.7. Agarose Gel Testing

The mechanical stability of nanoPt (deposited by 720 CV cycles) and IrO_x_ coatings (deposited by 60 min) on sPt-Ir and hPt-Ir substrates was also evaluated by agarose gel testing (Figure 1e) [23,24,25]. The agarose powder was dissolved in 95 °C water and stirred until a clear solution was obtained. The mixture was then cooled to 35 °C and transferred to a beaker and left to set overnight. The electrode was inserted into the gel 3 times at different positions with a depth of 30 mm each time via a micromanipulator driven at 2 mm/s, reaching a total distance of 180 mm. OM, CV and EIS characterizations were conducted before testing. After each insertion, the electrodes were rinsed and characterized by CV and EIS. The averages and error bars of three set of insertions were calculated for the results in EIS. After completing three sets of insertions, the microscopic morphology of the electrodes was characterized using SEM and OM. 

### 2.8. Cyclic Voltammetry Testing

Cyclic voltammetry testing was performed on electrodes with nanoPt deposited by 720 CV cycles and IrO_x_ coatings deposited by 60 min on sPt-Ir and hPt-Ir substrates. The test was conducted in the potential range of −0.6 to 0.8 V versus Ag/AgCl electrode at a constant scan rate of 100 mV/s in 0.01 M 1× PBS solution for 1500 cycles, as shown in Figure 1f [17]. Three samples of each type of electrode were tested. The CV and EIS performance and OM morphology of the electrodes were characterized before and after the test using the method described above. SEM and OM inspection were conducted at the end of the testing.

## 3. Results

### 3.1. Electrodeposition of NanoPt and IrO_x_ Coatings

**Figure 2:**
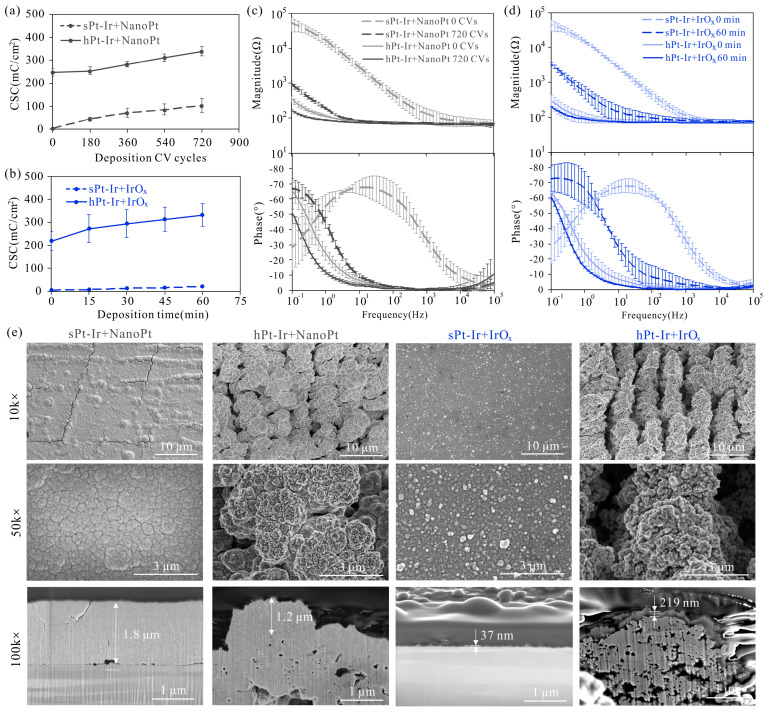
Electrochemical performance and surface morphology of electrodeposited nanoPt and IrO_x_ coatings on sPt-Ir and hPt-Ir substrates. The charge-storage capacity of (**a**) nanoPt and (**b**) IrO_x_ varied with deposition CV cycles or deposition time. Impedance magnitude and phase before and after adding (**c**) nanoPt (deposited by 720 CV cycles) and (**d**) IrO_x_ coatings (deposited by 60 min), (**e**) surface and cross-sectional morphology of nanoPt (deposited by 720 CV cycles) and IrO_x_ (deposited by 60 min) on sPt-Ir and hPt-Ir substrates. There were 3 samples of each kind of electrode.

**Figure 3:**
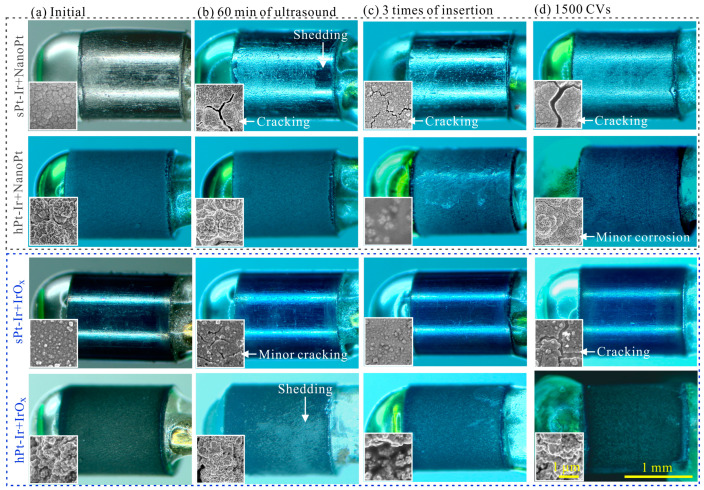
Surface morphology and failure summary of nanoPt and IrO_x_ coatings on sPt-Ir and hPt-Ir substrates (**a**) initially and after (**b**) ultrasonic, (**c**) agarose gel, and (**d**) cyclic voltammetry testing.

The electrochemical properties and surface morphology of nanoPt and IrO_x_ are shown in Figure 2 and Figure 3a. Both coatings exhibited an increased charge-storage capacity with increasing deposition CV cycles or deposition time, and the coatings prepared on the hPt-Ir substrates had a higher CSC than those on the sPt-Ir substrates (Figure 2a,b). The maximum CSC of both coatings on hPt-Ir exceeded 330 mC/cm^2^, which was more than 75 times that of the smooth Pt-Ir electrode (~4.3 mC/cm^2^). For the nanoPt coating, the growth rates of CSC versus deposition time were comparable on both substrates, while the IrO_x_ coating on the hierarchical substrate had a much higher CSC growth rate than that on the smooth substrate. As the CSC increased after deposition, the impedance magnitude of the electrodes decreased and the phase curve shifted to the lower frequencies (Figure 2c,d). The coatings on the hPt-Ir substrate had lower impedance magnitudes. As can be seen from SEM and FIB morphology in Figure 2e, the nanoPt coating was around 1~2 μm thick and showed pyramidal structures. Compared with the sPt-Ir substrates, the hPt-Ir substrates prevented cracking in the nanoPt coatings. The IrO_x_ coating was relatively thinner (below 250 nm) and therefore its surface profile was similar to that of the substrates. Nonetheless, nanoparticles could be found on the IrO_x_ coating surfaces.

**Figure 4:**
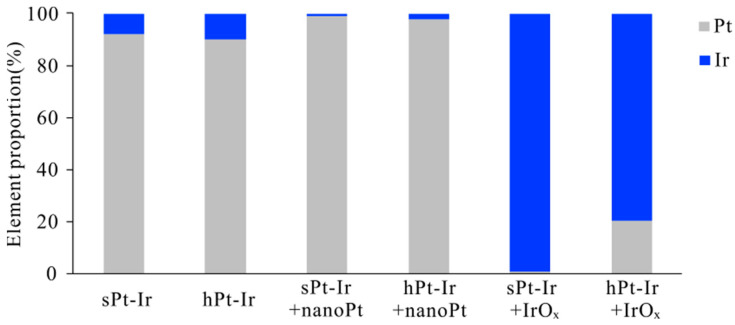
Platinum and iridium element proportion on sPt-Ir and hPt-Ir substrates with and without nanoPt and IrO_x_ coatings.

The surface elements before and after adding coatings are shown in Figure 4. The proportion of Pt and Ir on the smooth and hierarchical substrates was close to the element content of PtIr10 as designed (i.e., 90% platinum and 10% iridium). After adding the nanoPt coatings, the Pt content on both substrates exceeded 99%, indicating that the substrate surfaces were fully covered with the coatings. Following the deposition of IrO_x_ coatings, the Ir content exceeded 99% on the sPt-Ir but accounted for about 80% on the hPt-Ir substrate, suggesting that the coating was more fully covered on the former surface.

### 3.2. Mechanical Stability

**Figure 5:**
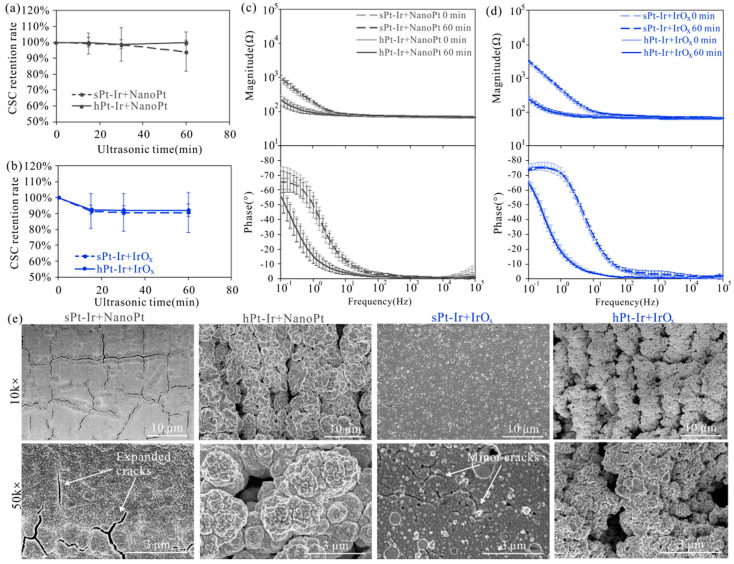
Ultrasonic testing results of electrodeposited nanoPt and IrO_x_ coatings. The charge-storage capacity retention rates of (**a**) nanoPt and (**b**) IrO_x_ varied with ultrasonic time. Impedance magnitude and phase of (**c**) nanoPt and (**d**) IrO_x_ coatings before and after ultrasonic testing. (**e**) Surface morphology of nanoPt and IrO_x_ on sPt-Ir and hPt-Ir substrates after 60 min of ultrasonic treatment. The were 3 samples of each kind of electrode.

The effects of ultrasonic treatment on the electrochemical properties and surface morphology of nanoPt and IrO_x_ coatings are shown in Figure 3b and Figure 5. Therein, CSC retention rate is defined as the ratio of CSC measured during testing to CSC value before testing. It was observed that as the duration of ultrasonic treatment increased, IrO_x_ coatings showed an obvious decrease in CSC at the time point of 15 min, earlier than that of nanoPt coatings (Figure 5a,b). Nevertheless, the CSC of both coatings on various substrates remained above 90% of their initial values after 60 min of treatment. Additionally, the impedance magnitude and phase of these coatings remained stable (Figure 5c,d). According to the SEM in Figure 5e, the nanoPt and IrO_x_ coatings on the sPt-Ir surfaces exhibited expanded and minor cracks, respectively. On the hPt-Ir substrates, the nanoPt coatings remained intact in both SEM and optical images (Figure 3b and Figure 5e). For the IrO_x_ coatings, it seemed to remain unchanged under SEM due to the ultrathin thickness of around tens of nanometers (Figure 5e), but some shedding could be observed in the optical picture in Figure 3b. The combined analysis of optical and SEM images could better characterize the change in coating morphology.

**Figure 6:**
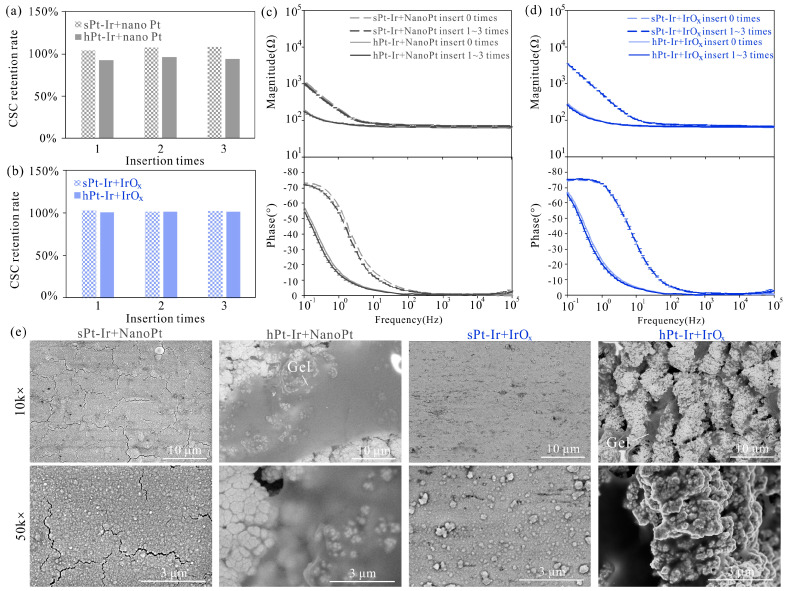
Agarose gel testing results of electrodeposited nanoPt and IrO_x_ coatings. The charge-storage capacity retention rates of (**a**) nanoPt and (**b**) IrO_x_ varied with insertion times. Impedance magnitude and phase of (**c**) nanoPt and (**d**) IrO_x_ coatings before and during 3 times of agarose gel testing. (**e**) Surface morphology of nanoPt and IrO_x_ on sPt-Ir and hPt-Ir substrates after agarose gel testing.

Agarose gel testing is another commonly used method to evaluate the mechanical stability of electrode coatings. After three insertions, the CSC of most coatings maintained close to 100% of their initial values except for the nanoPt coating on the hPt-Ir substrate (Figure 6a,b). Nevertheless, its CSC retained more than 94%. The impedance magnitude and phase curves of all coatings remained basically unchanged during three insertions (Figure 6c,d). As can be seen in the surface morphology, all coatings remained intact except some cracks in the nanoPt coatings on the sPt-Ir substrates (Figure 6e). Some gel adhered to the electrode surface, especially on the surface of the hPt-Ir substrate with nanoPt coating. This may be the reason for its CSC decrease. The results indicate that the electrodeposited nanoPt and IrO_x_ coatings on both substrates had good mechanical stability in the insertion process.

### 3.3. Electrochemical Stability

**Figure 7:**
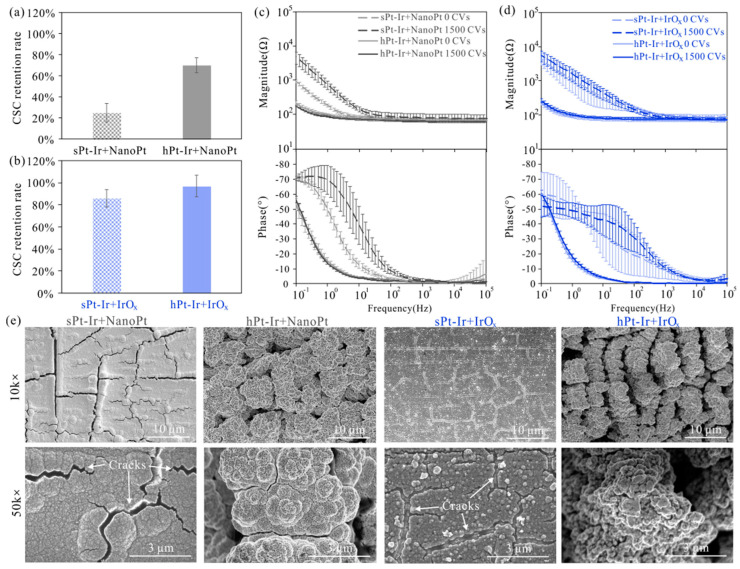
CV testing results of electrodeposited nanoPt and IrO_x_ coatings. The charge-storage capacity retention rates of (**a**) nanoPt and (**b**) IrO_x_ after 1500 CV cycles. Impedance magnitude and phase of (**c**) nanoPt and (**d**) IrO_x_ coatings before and after 1500 CV cycles. (**e**) Surface morphology of nanoPt and IrO_x_ coatings on sPt-Ir and hPt-Ir substrates after 1500 CV cycles. There were 3 samples of each kind of electrode.

Figure 7 shows the electrochemical performance and surface morphology of the two coatings after cyclic voltammetry testing. The CSC retention rates of the IrO_x_ coatings were higher than those of nano-Pt coatings after 1500 CV cycles (Figure 7a,b). Furthermore, the CSC retention rates of both coatings on the hPt-Ir substrates were higher than those on the sPt-Ir substrates. Meanwhile, their impedance magnitude remained generally stable compared to the increased impedance magnitudes of the coatings on the sPt-Ir substrates (Figure 7c,d). The increased variability of IrO_x_ coatings on sPt-Ir was considered to be related to the varying degrees of damage to the ultrathin coatings. Although the IrO_x_ on sPt-Ir was very thin, it had a significant effect on electrochemical impedance spectroscopy (Figure 2d). Therefore, the damage of the ultrathin coatings had a great effect on the EIS in Figure 7d. For the surface morphology, the nanoPt coatings on the smooth platinum-iridium substrate surface had many expanded cracks at 10k× magnification, while the same coatings on the hierarchical platinum-iridium substrate surface were relatively stable (Figure 7e). Notably, tiny gaps and micropores could be observed for the nanoPt coating on the hPt-Ir surface at 50k× magnification, which might have been the result of electrochemical corrosion during CV scanning. Nonetheless, the coating remained stably attached to the substrate without delamination. On the other hand, IrO_x_ coatings on the smooth substrate showed many cracks at 10k× magnification, while the coatings on the hierarchical substrates remained stable. Therefore, the hierarchical substrates can improve the stability for both nanoPt and IrO_x_ coatings under CV scanning.

## 4. Discussion

Electrochemically deposited platinum and iridium oxide coatings have attracted attention for application in implantable neural electrodes due to their convenient solution processing and in situ deposition manner [17]. However, the stability of these coatings is key to their long-term application. In this work, hierarchical platinum-iridium electrodes fabricated by femtosecond laser were used to improve the mechanical and electrochemical stability of both nanoPt and IrO_x_ coatings. The morphologies of these coatings before and after ultrasonic testing, agarose gel testing and 1500 cycles of CV scanning are summarized in Figure 3. The surface morphology and failure modes of the two coatings on stPt-Ir and hPt-Ir substrates were compared.

Electrodeposited nanoPt and IrO_x_ coatings exhibited distinct morphologies that were influenced by the solution composition and the deposition parameters employed (Figure 2e and Figure 3a). Electrochemical deposition techniques, which involve an electrochemical oxidation or reduction process, can control the nucleation and growth of nanocrystals. The formation of the nanoPt coating is achieved through an electrochemical reduction process. Boehler et al. [12] investigated the impact of various deposition parameters on the surface morphology of nanoPt coatings on platinum wire electrodes. They found that using the constant potential method resulted in flake-like coatings, while repeating the CV cycles led to a nanostructured film with pyramidal structures. The growth patterns can be explained by nucleation models for mass transport-controlled plating processes [26]. In this study, the CV method was selected for nanoPt deposition due to its ability to create a surface with homogenous coverage. Similar pyramidal structures can be found in Figure 2e. On the other hand, IrO_x_ coatings are formed by the anodic oxidation of ligands in an Ir complex compound [27,28]. Zeng et al. [15] examined the electrochemical properties and morphology of IrO_x_ coatings prepared by both CV and constant potential methods. In this work, the constant potential method was chosen in order to increase the deposition rate of IrO_x_ so that the deposition time could be set within an appropriate range. In this way, the IrO_x_ coating showed a similar morphology with nanoparticles reported in the literature [15]. Moreover, through the electrodeposition of nanoPt or IrO_x_ coatings on hierarchical structures, the charge-storage capacity of neural electrodes was enhanced to a higher level compared to similar materials in the literature [13] (Table 1).

The hierarchical substrate significantly improved the mechanical stability of the nanoPt coatings (Figure 3b,c). According to the CSC retention rate under ultrasonic testing, the nanoPt coating had better mechanical stability than the IrO_x_ coating (Figure 5a,b). This may be due to the stronger bonding between the nanoPt coating and the PtIr10 substrate, which is composed of 90% platinum and 10% iridium (Figure 4). As can be seen from the FIB cross-sectional profile, there was a clear boundary between the IrO_x_ coating and the substrate, while for the nanoPt coating, the boundary was hard to find, especially on the hPt-Ir substrates (Figure 2e). Moreover, the hierarchical substrate prevented the nanoPt coating from initial cracks. In a previous study [22], it was found that femtosecond laser direct writing could prepare hierarchical structures on Pt-Ir electrodes including micro-protrusions at a few micrometers and nanoparticles at a few hundreds of nanometers. Mechanisms such as rapid resolidification after ablation or redeposition of nanoparticles generated in ablation could give rise to the surface structures [31]. After electrodeposition, abundant mechanical anchoring formed between the hierarchical structures and the nanoPt coatings, which could help to improve the mechanical stability of the coatings [32]. However, for IrO_x_ coating on hPt-Ir substrate, there was a certain degree of shedding during ultrasonic treatment. It may be related to the inhomogeneous coverage surface of the coating at a high deposition rate. Nonetheless, the IrO_x_ coating still showed good stability in the agarose gel testing (Figure 6b,d,e). Optimizing the solute concentration and the electrodeposition parameters is a potential path to improving its mechanical stability [13,14,15].

CV scanning is a commonly used technique to evaluate the stability of electrode coatings under electrochemical conditions [17,23,33]. A 0.01 M PBS solution was used in the CV scanning to compare the coatings’ stability, due to its ease of preparation, stability, and reproducibility. Nonetheless, it has a higher ion concentration, and lacks bioactive substances such as proteins compared with in vivo conditions. Future testing in cerebrospinal fluid, perilymph, or degassed artificial interstitial fluid will help to better simulate the in vivo working environment [34]. In addition, for practical applications, electrodes are designed to be used over a few days [35] to up to more than 10 years [36]. In order to work safely over a longer period of time, the coating stability is a critical issue. Their performance in an in vivo environment should be carefully evaluated to ensure security during treatment.

In this study, the hierarchical Pt-Ir substrates prepared by femtosecond laser direct writing improved the stability of nanoPt and IrO_x_ coatings during CV scanning without the need for corrosive reagents (Figure 3d and Figure 7). The enhanced mechanical anchoring provided by the hierarchical platinum-iridium substrates could explain the improved electrochemical stability of the coatings [37]. Therein, the IrO_x_ coatings demonstrated a higher CSC retention rate and better stability in the morphology than the nanoPt coatings. This may have resulted from its ability to inject charge via a fast, reversible faradaic reaction involving reduction and oxidation between Ir^3+^ and Ir^4+^ states in the oxide [38]. Notably, the nanoPt coating on hPt-Ir experienced a decrease in CSC and showed tiny gaps and micropores, possibly due to microcorrosion during the CV cycles (Figure 7e). Generally, CV scanning provides a tougher electrochemical condition than clinical biphasic pulse stimulation [39], which may accelerate platinum dissolution. The dissolution of platinum can be quantified by measuring the ion concentration in the solution using inductively coupled plasma mass spectrometry (ICP-MS) or the mass change in the electrodes using electrochemical quartz crystal microbalance (EQCM). Wissel et al. [40] found dissolved platinum below a certain concentration did not cause toxicity to surrounding inner ear tissue in a cell culture model. Further, Shepherd et al. [41] found that long-term electrical stimulation at high-charge density in rat cochlear caused platinum electrode corrosion, but it did not result in neurological function loss or change. Additionally, the protein adsorption on the electrode was found to reduce the extent of Pt dissolution in vivo [42]. Nonetheless, the quantity of Pt dissolved during electrochemical testing in vitro and the stability of nanoPt electrodes under long-term electrical stimulation in vivo deserve further investigation in future work.

Stable nanoPt and IrO_x_ coatings not only improve neural electrical stimulation and recording performance, but also have the potential for in vivo neurobiochemical detection. The nanoPt coatings on the surface of the hierarchical Pt-Ir substrate significantly increase the electrochemical active area, which could be used for the non-enzymatic detection of glucose [17,43], hydrogen peroxide [44], and dissolved oxygen [44,45]. In addition, an IrO_x_ coating is being considered as a reference electrode candidate for in vivo biosensing due to its biocompatibility [46]. Ag/AgCl reference electrodes commonly used lack the in vivo potential stability required for long-term measurements, and their cytotoxicity affects animal experiments and human implantation [47,48]. The biocompatibility of IrO_x_ minimizes the disruption of proton balance to maintain constant potential [46], and the coating stability could help keep its function. Therefore, the stable nanoPt and IrO_x_ coatings could benefit the development of multimodal neural electrodes.

## 5. Conclusions

NanoPt and IrO_x_ coatings on hierarchical platinum-iridium substrates exhibited highly improved charge-storage capacity and decreased impedance magnitude. The nanoPt coating on the hierarchical substrate exhibited good stability under both ultrasonic testing of 1 h and insertion testing into agarose gel to a total distance of 180 mm. Although partial peeling off of iridium oxide coating on hPt-Ir substrates was observed during ultrasonic testing, it remained stable in the agarose gel testing. Additionally, the hierarchical platinum-iridium substrate improved the CV stability for both coatings. Therein, iridium oxide showed better stability than nanoPt coating in the CV scanning. The improved coating stability and electrochemical properties could not only enhance neural electrical stimulation and recording performance but also facilitate the development of in vivo biochemical sensing functionalities.

## Figures and Tables

**Table 1:** Comparison of charge-storage capacity of platinum and iridium oxide electrodes in the literature.

Materials	Increased Times of CSC	References
NanoPt	32.5	[12]
Pt nanograss	40	[29]
Roughed Pt	44	[30]
3D IrO_x_/Pt	60	[15]
NanoPt or IrO_x_ on hierarchical Pt-Ir	75	This work

## Data Availability

Data are contained within the article.

## References

[1] Wong J.K., Mayberg H.S., Wang D.D., Richardson R.M., Halpern C.H., Krinke L., Arlotti M., Rossi L., Priori A., Marceglia S. (2022). Proceedings of the 10th annual deep brain stimulation think tank: Advances in cutting edge technologies, artificial intelligence, neuromodulation, neuroethics, interventional psychiatry, and women in neuromodulation. Front. Hum. Neurosci..

[2] Carron R., Roncon P., Lagarde S., Dibué M., Zanello M., Bartolomei F. (2023). Latest views on the mechanisms of action of surgically implanted cervical vagal nerve stimulation in epilepsy. Neuromodul. Technol. Neural Interface.

[3] Lorach H., Galvez A., Spagnolo V., Martel F., Karakas S., Intering N., Vat M., Faivre O., Harte C., Komi S. (2023). Walking naturally after spinal cord injury using a brain-spine interface. Nature.

[4] Wu K.Y., Mina M., Sahyoun J., Kalevar A., Tran S.D. (2023). Retinal prostheses: Engineering and clinical perspectives for vision restoration. Sensors.

[5] Borda E., Ghezzi D. (2022). Advances in visual prostheses: Engineering and biological challenges. Prog. Biomed. Eng..

[6] Eggers T., Kilgore J., Green D., Vrabec T., Kilgore K., Bhadra N. (2021). Combining direct current and kilohertz frequency alternating current to mitigate onset activity during electrical nerve block. J. Neural Eng..

[7] Jones M.G., Rogers E.R., Harris J.P., Sullivan A., Ackermann D.M., Russo M., Lempka S.F., Mcmahon S.B. (2021). Neuromodulation using ultra low frequency current waveform reversibly blocks axonal conduction and chronic pain. Sci. Transl. Med..

[8] Zeng Q., Huang Z. (2023). Challenges and opportunities of implantable neural interfaces: From material, electrochemical and biological perspectives. Adv. Funct. Mater..

[9] Zheng X.S., Tan C., Castagnola E., Cui X.T. (2021). Electrode Materials for Chronic Electrical Microstimulation. Adv. Healthc. Mater..

[10] Dalrymple A.N., Robles U.A., Huynh M., Nayagam B.A., Green R.A., Poole-Warren L.A., Fallon J.B., Shepherd R.K. (2020). Electrochemical and biological performance of chronically stimulated conductive hydrogel electrodes. J. Neural Eng..

[11] Basova T.V., Vikulova E.S., Dorovskikh S.I., Hassan A., Morozova N.B. (2021). The use of noble metal coatings and nanoparticles for the modification of medical implant materials. Mater. Des..

[12] Boehler C., Vieira D.M., Egert U., Asplund M. (2020). NanoPt—A nanostructured electrode coating for neural recording and microstimulation. ACS Appl. Mater. Interfaces.

[13] Zeng Q., Yu S., Fan Z., Huang Y., Song B., Zhou T. (2022). Nanocone-array-based platinum-iridium oxide neural microelectrodes: Structure, electrochemistry, durability and biocompatibility study. Nanomaterials.

[14] Zeng Q., Xia K., Zhang Y., Wu T. (2019). Well controlled 3D iridium oxide/platinum nanocomposites with greatly enhanced electrochemical performances. Adv. Mater. Interfaces.

[15] Zeng Q., Huang Z., Cai G., Wu T. (2021). Platinum nanocrystal assisted by low-content iridium for high-performance flexible electrode: Applications on neural interface, water oxidation, and anti-microbial contamination. Adv. Mater. Interfaces.

[16] Kim R., Nam Y. (2015). Electrochemical layer-by-layer approach to fabricate mechanically stable platinum black microelectrodes using a mussel-inspired polydopamine adhesive. J. Neural Eng..

[17] Ivanovskaya A.N., Belle A.M., Yorita A.M., Qian F., Chen S., Tooker A., Lozada R.G.I., Dahlquist D., Tolosa V. (2018). Electrochemical roughening of thin-film platinum for neural probe arrays and biosensing applications. J. Electrochem. Soc..

[18] Zhang K., Deng J., Guo X., Sun L., Lei S. (2018). Study on the adhesion and tribological behavior of PVD TiAlN coatings with a multi-scale textured substrate surface. Int. J. Refract. Hard Met..

[19] Park J., Park B., Son Y.J., Lee S.H., Um S., Kim Y., Ok M., Sun J., Han H., Jeon H. (2021). Femtosecond laser-mediated anchoring of polymer layers on the surface of a biodegradable metal. J. Magnes. Alloys.

[20] Zhang P., Zou X., Zhang S., Xia C., Liang C., Liu N., Wang H. (2021). Improve the binding force of PEEK coating with Mg surface by femtosecond lasers induced micro/nanostructures. J. Mater. Sci..

[21] Li L., Jiang C., Duan W., Wang Z., Zhang F., He C., Long T., Li L. (2022). Electrochemical and biological performance of hierarchical platinum-iridium electrodes structured by a femtosecond laser. Microsyst. Nanoeng..

[22] Li L., Jiang C., Li L. (2022). Hierarchical platinum-iridium neural electrodes structured by femtosecond laser for superwicking interface and superior charge storage capacity. Bio-Des. Manuf..

[23] Ramesh V., Stratmann N., Schaufler V., Angelov S.D., Nordhorn I.D., Heissler H.E., Martínez-Hincapié R., Čolić V., Rehbock C., Schwabe K. (2022). Mechanical stability of nano-coatings on clinically applicable electrodes, generated by electrophoretic deposition. Adv. Healthc. Mater..

[24] Hyakumura T., Aregueta-Robles U., Duan W., Villalobos J., Adams W.K., Poole-Warren L., Fallon J.B. (2021). Improving deep brain stimulation electrode performance in vivo through use of conductive hydrogel coatings. Front. Neurosci..

[25] Boehler C., Oberueber F., Stieglitz T., Asplund M. Nanostructured platinum as an electrochemically and mechanically stable electrode coating. Proceedings of the 39th Annual International Conference of the IEEE Engineering in Medicine and Biology Society (EMBC).

[26] Plyasova L.M., Molina I.Y., Gavrilov A.N., Cherepanova S.V., Cherstiouk O.V., Rudina N.A., Savinova E.R., Tsirlina G.A. (2006). Electrodeposited platinum revisited: Tuning nanostructure via the deposition potential. Electrochim. Acta.

[27] Obradović M.D., Balanč B.D., Lačnjevac U., Gojković S.L. (2021). Electrochemically deposited iridium-oxide: Estimation of intrinsic activity and stability in oxygen evolution in acid solution. J. Electroanal. Chem..

[28] Kötz R., Neff H., Stucki S. (1984). Anodic iridium oxide films: XPS-studies of oxidation state changes and O_2_-Evolution. J. Electrochem. Soc..

[29] Boehler C., Stieglitz T., Asplund M. (2015). Nanostructured platinum grass enables superior impedance reduction for neural microelectrodes. Biomaterials.

[30] Weremfo A., Carter P., Hibbert D.B., Zhao C. (2015). Investigating the interfacial properties of electrochemically roughened platinum electrodes for neural stimulation. Langmuir.

[31] Vorobyev A.Y., Guo C. (2013). Direct femtosecond laser surface nano/microstructuring and its applications. Laser Photonics Rev..

[32] Pranti A.S., Schander A., Bödecker A., Lang W. (2018). PEDOT: PSS coating on gold microelectrodes with excellent stability and high charge injection capacity for chronic neural interfaces. Sens. Actuators B Chem..

[33] Fan P., Song Y., Lu B., Wang Y., Dai Y., Xie J., He E., Xu Z., Yang G., Mo F. (2022). PtNPs/PEDOT:PSS-modified microelectrode arrays reveal electrophysiological activities of different neurons in medial amygdala of mice under innate fear. Front. Neurosci..

[34] Harris A.R., Newbold C., Stathopoulos D., Carter P., Cowan R., Wallace G.G. (2022). Comparison of the in vitro and in vivo electrochemical performance of bionic electrodes. Micromachines.

[35] Miller C., Schatmeyer B., Landazuri P., Uysal U., Nazzaro J., Kinsman M.J., Camarata P.J., Ulloa C.M., Hammond N., Pearson C. (2021). SEEG for expansion of a surgical epilepsy program: Safety and efficacy in 152 consecutive cases. Epilepsia Open.

[36] Bove F., Mulas D., Cavallieri F., Castrioto A., Chabardès S., Meoni S., Schmitt E., Bichon A., Di Stasio E., Kistner A. (2021). Long-term outcomes (15 years) after subthalamic nucleus deep brain stimulation in patients with Parkinson disease. Neurology.

[37] Ganji M., Hossain L., Tanaka A., Thunemann M., Halgren E., Gilja V., Devor A., Dayeh S.A. (2018). Monolithic and scalable Au nanorod substrates improve PEDOT-metal adhesion and stability in neural electrodes. Adv. Healthc. Mater..

[38] Cogan S.F. (2008). Neural stimulation and recording electrodes. Annu. Rev. Biomed. Eng..

[39] Boehler C., Oberueber F., Schlabach S., Stieglitz T., Asplund M. (2017). Long-term stable adhesion for conducting polymers in biomedical applications: IrOx and nanostructured platinum solve the chronic challenge. ACS Appl. Mater. Inter..

[40] Wissel K., Brandes G., Pütz N., Angrisani G.L., Thieleke J., Lenarz T., Durisin M. (2018). Platinum corrosion products from electrode contacts of human cochlear implants induce cell death in cell culture models. PLoS ONE.

[41] Shepherd R.K., Carter P.M., Enke Y.L., Wise A.K., Fallon J.B. (2019). Chronic intracochlear electrical stimulation at high charge densities results in platinum dissolution but not neural loss or functional changes in vivo. J. Neural Eng..

[42] Clark G.M., Clark J., Cardamone T., Clarke M., Nielsen P., Jones R., Arhatari B., Birbilis N., Curtain R., Xu J. (2014). Biomedical studies on temporal bones of the first multi-channel cochlear implant patient at the University of Melbourne. Cochlear Implants Int..

[43] Mccormick W., Mccrudden D. (2020). Development of a highly nanoporous platinum screen-printed electrode and its application in glucose sensing. J. Electroanal. Chem..

[44] Weltin A., Kieninger J., Urban G.A., Arndt S., Rosskothen-Kuhl N. Cochlear implant electrodes as electrochemical sensors in vivo. Proceedings of the 10th International IEEE/EMBS Conference on Neural Engineering (NER).

[45] Dong Q., Sun X., He S. (2021). Iridium oxide enabled sensors applications. Catalysts.

[46] Seaton B.T., Heien M.L. (2021). Biocompatible reference electrodes to enhance chronic electrochemical signal fidelity in vivo. Anal. Bioanal. Chem..

[47] Seaton B.T., Hill D.F., Cowen S.L., Heien M.L. (2020). Mitigating the effects of electrode biofouling-induced impedance for improved long-term electrochemical measurements in vivo. Anal. Chem..

[48] Robbins E.M., Castagnola E., Cui X.T. (2022). Accurate and stable chronic in vivo voltammetry enabled by a replaceable subcutaneous reference electrode. iScience.

